# Molecular and Functional Profiling of the Polyamine Content in Enteroinvasive *E. coli* : Looking into the Gap between Commensal *E. coli* and Harmful *Shigella*

**DOI:** 10.1371/journal.pone.0106589

**Published:** 2014-09-05

**Authors:** Rosaria Campilongo, Maria Letizia Di Martino, Lucia Marcocci, Paola Pietrangeli, Adriano Leuzzi, Milena Grossi, Mariassunta Casalino, Mauro Nicoletti, Gioacchino Micheli, Bianca Colonna, Gianni Prosseda

**Affiliations:** 1 Istituto Pasteur-Fondazione Cenci Bolognetti, Dipartimento di Biologia e Biotecnologie “C. Darwin”, Sapienza Università di Roma, Roma, Italy; 2 Dipartimento di Scienze, Università Roma Tre, Roma, Italy; 3 Dipartimento di Biochimica, Sapienza Università di Roma, Roma, Italy; 4 Dipartimento di Scienze Sperimentali e Cliniche, Università G. D'Annunzio, Chieti, Italy; 5 Istituto di Biologia, Medicina Molecolare e NanoBiotecnologie, Consiglio Nazionale delle Ricerche, Roma, Italy; Rockefeller University, United States of America

## Abstract

Polyamines are small molecules associated with a wide variety of physiological functions. Bacterial pathogens have developed subtle strategies to exploit polyamines or manipulate polyamine-related processes to optimize fitness within the host. During the transition from its innocuous *E. coli* ancestor, *Shigella*, the aetiological agent of bacillary dysentery, has undergone drastic genomic rearrangements affecting the polyamine profile. A pathoadaptation process involving the *speG* gene and the *cad* operon has led to spermidine accumulation and loss of cadaverine. While a higher spermidine content promotes the survival of *Shigella* within infected macrophages, the lack of cadaverine boosts the pathogenic potential of the bacterium in host tissues. Enteroinvasive *E. coli* (EIEC) display the same pathogenicity process as *Shigella*, but have a higher infectious dose and a higher metabolic activity. Pathoadaption events affecting the *cad* locus have occurred also in EIEC, silencing cadaverine production. Since EIEC are commonly regarded as evolutionary intermediates between *E. coli* and *Shigella*, we investigated on their polyamine profile in order to better understand which changes have occurred along the path to pathogenicity. By functional and molecular analyses carried out in EIEC strains belonging to different serotypes, we show that *speG* has been silenced in one strain only, favouring resistance to oxidative stress conditions and survival within macrophages. At the same time, we observe that the content of spermidine and putrescine, a relevant intermediate in the synthesis of spermidine, is higher in all strains as compared to *E. coli*. This may represent an evolutionary response to the lack of cadaverine. Indeed, restoring cadaverine synthesis decreases the expression of the *speC* gene, whose product affects putrescine production. In the light of these results, we discuss the possible impact of pathoadaptation events on the evolutionary emergence of a polyamine profile favouring to the pathogenic lifestyle of *Shigella* and EIEC.

## Introduction

*Escherichia coli* is not only a harmless commensal of the human and animal intestine, but also a major cause of morbidity and mortality [1], [2]. As in many other bacterial pathogens, the evolution of *E. coli* towards pathogenic phenotypes has been determined mainly by two mechanisms: the acquisition of virulence genes and the loss or modification of genes of the core genome [3]. *E. coli* acquires virulence determinants by horizontal gene transfer as parts of plasmids, bacteriophages, transposons or pathogencity islands, and this process plays a crucial role in the colonization of a new host environment and in the successful establishment of a pathogenic lifestyle [4], [5]. Genome analyses clearly confirm that also the loss of genes by means of so-called pathoadaptive mutations has strongly contributed to the emergence of pathogenic *E. coli* strains [6], [7].

On the basis of specific virulence factors and pathogenicity processes, pathogenic *E. coli* have been subdivided into different pathotypes, including intestinal and extraintestinal strains [1]. Among intestinal pathogenic *E. coli*, enteroinvasive *E. coli* (EIEC) are intracellular pathogens causing a severe enteric syndrome in humans, mainly in developing countries [5]. The pathogenesis of EIEC strains is based on their capacity to invade cells of the colonic epithelium, replicate intracellularly and spread to adjacent cells causing inflammatory destruction of the intestinal epithelial barrier. The mechanism is similar to that of *Shigella*, the causative agent of bacillary dysentery [8] and EIEC strains are therefore included in the same pathotype as *Shigella* [1]. However, EIEC do not display the full set of characters that define *Shigella* nor they underwent the extensive gene decay observed in *Shigella* [9]. Molecular analyses confirm that EIEC strains are widely distributed among *E. coli* phylogenetic groups and generally correspond to bioserotypes found in a dozen of *E. coli* serogroups [10], [11], [12]. Many EIEC strains have *Shigella*-like features: they can be Lac^−^, non-motile, low-level indole-producing, and (at least in some cases) they carry the typical *Shigella* O-antigen. On the other hand, in contrast to *Shigella*, EIEC have a higher infectious dose and a higher metabolic activity, retaining the ability to catabolize substrates widely used by *E. coli* [10], [13], [14].

It is widely acknowledged that the major event that gave rise to the *Shigella/*EIEC pathotype has been the acquisition of a large virulence plasmid (pINV) coding for the invasion plasmid antigens (Ipa proteins), for their Type III secretion system (T3SS), and for many other genes involved in invasion, survival and intracellular spread [15], [16], [17], [18]. The expression of pINV genes is affected by multiple environmental stimuli acting through a regulatory cascade which involves nucleoid proteins, specific regulators and sRNAs encoded by both, pINV and the chromosome [19], [20], [21]. A complementary yet significant step towards the pathogenic lifestyle has been the inactivation of several chromosomal genes which negatively interfere with the expression of virulence factors required for survival within the host. This process, commonly referred to as pathoadaptation, has been mainly studied in *Shigella* spp. [6], [7]. The antivirulence loci identified so far in *Shigella* encode a broad spectrum of functions, confirming that adaptation to the new host environments is the result of a long and ordered process targeting core genome determinants.

Among pathoadaptive mutations, a paradigmatic case is represented by those affecting the polyamine content of *Shigella* [22], [23]. Polyamines are small polycationic molecules found in almost all cells and are associated with a wide variety of physiological functions, including translation, gene regulation, stress resistance, cell proliferation, and differentiation [24], [25]. Considerable evidence has recently built up that bacteria have evolved mechanisms to turn polyamines to their own advantage in order to increase their fitness within the host. In particular, major polyamines (cadaverine, spermidine, putrescine) have been shown to optimize virulence processes and intracellular survival rates in several human pathogens [26], [27]. In this respect, *Shigella* exhibits a peculiarity since polyamines have antagonistic effects on the invasive process: while higher spermidine levels correlate with an increased survival of *Shigella* during the infection of macrophages [22], the lack of cadaverine production increases the pathogenic potential of the bacterium in host tissues [23]. The higher level of spermidine is determined by the absence of spermidine acetyltransferase (SAT), the enzyme (encoded by the *speG* gene) which converts spermidine into its inert form, N-acetylspermidine. Cadaverine, whose synthesis depends on the *cad* gene [28], normally blocks the release of *Shigella* into the cytoplasm of the infected cells and inhibits the migration of polymorphonuclear leukocytes across the intestinal epithelium [6], [29]. The lack of functional *cad* and *speG* genes is the result of a convergent evolution, which banks on inactivating events ranging from point mutations to large deletions [23], [30], [31].

Besides the acquisition of the pINV plasmid, EIEC and *Shigella* share additional pathoadaptive mutations, e.g. the lack of the OmpT protease [32] and the loss of cadaverine production [33]. Another typical pathoadaptive mutation observed in *Shigella* is the requirement for exogenous nicotinic acid, due to inactivation of the *nad* genes [34]. It has been shown recently that, as opposed to *Shigella*, the inability to synthesize nicotinic acid is not a generalized feature among EIEC strains [35]. This agrees with the view that EIEC may represent an evolutionary intermediate in the transition towards a full-blown phenotype, with some mutational events still confined to *Shigella*.

In the present study, we have investigated on the polyamine profile in EIEC in order to understand whether, besides *cad* genes, also other genes involved in polyamine metabolism had been lost during pathoadaptation. Moreover, we asked if and how the loss of cadaverine may have affected the expression of other genes involved in the synthesis of polyamines. The results we present reveal that EIEC have an intermediate polyamine content as compared to *E. coli* and *Shigella* and that cadaverine negatively affects the synthesis of putrescine. All together our observations contribute to reconstruct the evolutionary events that have determined the emergence in *Shigella* of a polyamine profile well suited to an invasive lifestyle.

## Materials and Methods

### Bacterial strains and plasmids

Bacterial strains are listed in Table 1. *E. coli* ULS655 carries a deletion of the *cadC* gene [36]. Strain ULS86, carrying a deletion of the entire *cadBA* operon, and strains EIEC HN280 DG and 53638 DG, carrying a *speG* deletion, were constructed using the one-step method of gene inactivation [37] by transforming MG1655 pKD46, HN280 pKD46 and 53638 pKD46 with amplicons obtained using the plasmid pKD13 as template and the oligo pair *caf*/*car* (*cadBA* deletion) or *dgf*/*dgr* (*speG* deletion) (Table S1). Under the experimental conditions used, no differences in growth rate were observed between EIEC strains and their *speG* derivatives.

**Table 1:** Bacterial strains used in this study.

Bacterial strains	Serotype	Relevant Features	Defective gene in the *cad* locus	Source or reference
**EIEC**				
** HN280**	O135	Lac^−^, pINV (260 kb), pCRY (160 kb), LDC^−b^	*cadC*	Prosseda et al., 2006
** 4608**	O143	Lac+, pINV (250 kb), LDC-	*cadC*	WRAIR^c^
** 53638**	O144	Lac^−^, pINV (250 kb), pCRY (140 kb), LDC^−^	*cadC*	WRAIR^c^
** 13.80**	O124	Lac^−^, pINV (240 kb), LDC^−^	*cadC cadBA*	IPC^d^
** 6.81**	O115	Lac^−^, pINV (250 kb), pCRY (160 kb), LDC^−^	*cadC cadBA*	IPC^d^
** HN280 DG**	O135	HN280 derivative *ΔspeG*	*cadC*	This study
** 53638 DG**	O144	53638 derivative *ΔspeG*	*cadC*	This study
***S.flexneri***				
** M90T**	5	pINV (213 kb), pCRY (2.1 and 6.1 kb), LDC^−^	*cadC cadBA*	Sansonetti et al., 1982
***E. coli K-12***				
** MG1655**		*E. coli* K12, F^−^ *ilvG rfb-50 rph-1*; LDC^+^		ATCC^e^
** ULS655**		MG1655 derivative *ΔcadC*, Km^r^, LDC^−^	*cadC*	Casalino et al., 2010
** ULS86**		MG1655 derivative *ΔcadBA*, Km^r^, LDC^−^	*cadBA*	This study
** DH10b**		F^−^ *recA*1 Δ*lac*X74 *gal*K16 *ara*D139 *rpsL*		Invitrogen-Life Technologies, Inc

a All the strains are designed by their original laboratory name.

b LDC, lysine decarboxylase.

c WRAIR, Walter Reed Army Institute of Research.

d IPC, Institut Pasteur Collection.

e ATCC, American Type Culture Collection.

Plasmid pCC55 is a pACYC184 derivative carrying the *cadC* gene of MG1655 [33]. pULS37 and pULS13 are pACYC184 derivatives containing the *E. coli speG* gene under the control of its regulatory region or of the P*tac* promoter, respectively [22]. Plasmids containing the *ynfB*-*speG* locus of EIEC strains (pG13.80, pG6.81, pG4608, pG53630, and pG280) were constructed by cloning into pGEM-T Easy amplicons obtained using the *pgf*/*ygt* oligo pair (Table S1) and the corresponding EIEC genomic DNA as template. DH10b was used as recipient in cloning experiments.

Bacterial cells were routinely grown at 37°C in Luria-Bertani (LB) broth. When required cells were grown in polyamine free M9 complete medium (M9 minimal medium supplemented with 10 µg/ml thiamine, 0.2% glucose, 0.5% casamino acids and 10 µg/ml nicotinic acid). Solid media contained 1.6% agar. When required, antibiotics were included at the following concentrations: ampicillin (Ap), 100 µg/ml; chloramphenicol (Cm) 30 µg/ml; kanamycin (Km) 30 µg/ml, streptomycin (Sm) 50 µg/ml; tetracycline, (Tc) 5 µg/ml.

### General molecular procedures

DNA purification, transformation, restriction, electrophoresis, amplification and purification of DNA fragments were carried out as described previously [22], [38]. Pfu Taq DNA polymerase was used to obtain longer transcripts and high fidelity. All oligonucleotides used in this study are listed in Table S1 and have been designed mainly on the basis of the genomic sequence of the *Escherichia coli* K-12 MG1655 [39].

### Real time PCR

Total RNA purification and cDNA synthesis was performed as previously described [40]. Real time PCR was performed using a 30 µl reaction mix containing 2 µl cDNA. At least three wells were run for each sample. The amount of *speG* and *speC* transcripts was analysed using the 2^−ΔΔCt^ method [41] and the results were indicated as n-fold increase relative to the reference sample. The ΔCt-values in the Student's t test have been considered to determine whether datasets of relative gene expression were significantly different from those in a chosen calibrator. Primers for the *nusA* transcript, used as endogenous control, and for the above-mentioned transcripts were experimentally validated for suitability to the 2^−ΔΔCt^ method. The following oligos (Table S1) were used: *rgf*/*rgr* for *speG*, *scf*/*scr* for *speC*, and *nusAF*/*nusAR* for *nusA*.

### Primer extension

Total RNA from EIEC strains HN280, 13.80 and 4608 grown to OD_600_ 0.6 was extracted by a modified hot-phenol method and quantified spectrophotometrically as described [42]. The primer (*peg*) was 5′-end labelled with [γ-^32^P]dATP using T4 polynucleotide kinase and hybridized with 50 µg total RNA as previously described [43]. Reverse transcription experiments were carried out and the resulting cDNAs were run on denaturing 6% polyacrylamide gels, along with a sequencing ladder that was generated by using the same primer and HN280 and 4608 DNAs as templates. Sequencing reactions were performed with [α-^32^P]dATP.

### Polyamine quantification

Bacteria were grown in M9 complete medium to OD_600_ 0.7-0.8, centrifuged and resuspended in PBS. Cells were disrupted by sonication and polyamines were extracted from the lysate with 3% percloric acid containing 5 mM 1,6-diaminehexane as a polyamine internal standard. After derivatization with dansylchloride, the simultaneous fluorimetric determination of intracellular polyamines was performed by reverse-phase high-performance liquid chromatography and polyamines were quantified as described previously [44]. The polyamine concentration in total cellular homogenates was normalized to cell number and expressed as nmol/10^8^ cells.

### Bacterial sensitivity to oxidative stress

The susceptibility to oxidative stress was evaluated by analysing the survival of EIEC HN280 and 53638 strains and their derivatives after exposure to H_2_O_2_ in liquid cultures. To this end, 15 ml of bacterial cultures grown in M9 complete medium to OD_600_ 0.7 were centrifuged and the pellets were suspended in 1 ml PBS. An equal volume of PBS containing 20 mM H_2_O_2_ was added and the mix was incubated at 37°C for 1 h. The reaction was stopped by adding catalase to 0.1 mg/ml. The number of bacteria surviving oxidative stress was quantified by plating aliquots on LB agar. Survival percentages were calculated by comparison with the corresponding untreated strains.

### Ornithine decarboxylase activity assays

Monitoring of ornithine decarboxylase (L-ornithine decarboxylase, ODC) activity was performed according to the Ngo method [45]. Bacterial cultures were grown overnight, diluted in fresh M9 complete medium and allowed to grow to OD_600_ 0.7–0.8. 10 ml for each culture were centrifuged and pellets suspended in 1 ml PBS. 200 U of DNase and 10 µg/ml of RNase A were added to the samples and, after 30 minutes on ice, sonicated. Lysates were centrifuged at 14000 rpm for 15 minutes and supernatants were filtered (0.22 µm) and then dialyzed against 150 mM phosphate buffer. 100 µl aliquots were incubated at 37°C for 30 minutes in 400 µl reaction mixture containing 3 mM L-ornithine, 75 nM pyridoxal phosphate (PLP), 1.5 mM EDTA and 2.5 mM β-mercaptoethanol. Reactions were stopped by adding 20 µl 70% perchloric acid. Samples were centrifuged at 14000 rpm for 15 minutes and 200 µl of the supernatant were derivatized with dansyl chloride [46]. 10 µl of each sample were used to perform the TLC assay on Silica gel. The TLC separation was performed using chloroform:triethylamine (25∶2) as solvent. Finally, bands corresponding to putrescine were scraped from the plate and, after ethyl acetate extraction, fluorescence was measured on supernatants using a multilabel counter. Protein concentrations were determined by the Bradford method.

### Cultures of macrophages and bacterial infection

The murine macrophage-like J774 cells (ATCC, Manassas, VA) were cultured in RPMI 1640 medium containing 10% heat-inactivated fetal bovine serum, 2 mM L-glutamine and penicillin-streptomycin at 37°C in a humidified 5% CO_2_ atmosphere. For bacterial infection, 4×10^5^ cells per well were seeded in 12-well tissue culture plates and grown overnight. Before infection, host cells were cultured with serum-free, antibiotic-free medium for 1 hour. In order to produce a competitive infection, J774 cells were simultaneously infected with HN280 pACYC184 and HN280 pULS13 at a multiplicity of infection of 100 and centrifuged 10 min at 700 *g*. After a 30 min incubation at 37°C (time 0) cells were extensively washed with PBS and fresh cell culture medium containing 10 µg/ml of gentamicin was added to kill extracellular bacteria. The cell/bacteria mixture was further incubated at 37°C for the indicated time. To determine the number of intracellular bacteria, the cells were washed once with PBS and lysed by adding 0.5 ml of 1% Triton X-100 in PBS to each well for 5 min. Samples were mixed, diluted and plated onto LB agar to determine the number of CFU recovered from the lysate. The number of intracellular bacteria at different time points was compared to bacteria recovered at time zero. To calculate the competitive index (C.I.), the ratios of strains HN280 pULS13/HN280 pACYC184 recovered from the infected cultures were determined and normalized by dividing by the corresponding ratio in the initial inoculum.

### Nucleotide sequence accession numbers

DNA from plasmids pG13.80, pG6.81, pG4608, pG53630, and pG280 was used as template for sequencing the *ynfB-speG* locus. The sequence data were compared to known nucleotide and protein sequences using the BLAST server (National Center of Biotechnology Information, Bethesda, Md.). The *ynfB-speG* sequences were deposited at GeneBank under accession numbers KJ825879; KJ825880; KJ825881; KJ825882; and KJ825883.

## Results

### The polyamine content of EIEC strains

Enteroinvasive *Escherichia coli* (EIEC) do not represent a homogeneous group of pathogenic *E. coli*. Indeed, EIEC strains differ widely with respect to biochemical features, serotype, and plasmid content. Molecular phylogenetic analyses show that during evolution EIEC have derived several times independently from *E. coli* [10], [12]. In analogy to *Shigella*, all EIEC have lost the ability to synthetize cadaverine [33]. In a previous study [22] we have shown that *Shigella* has also lost *speG*, the gene encoding spermidine acetylase (SAT, an enzyme catalyzing the synthesis of N-acetylspermidine from spermidine), and that the lack of SAT induces a beneficial accumulation of intracellular spermidine favouring bacterial survival under oxidative stress conditions.

Since EIEC and *Shigella* display essentially the same pathogenicity mechanism and belong to the same pathotype, we asked whether they also share the same polyamine profile. To this end, we analysed a set of five invasive EIEC strains (HN280, 13.80, 4608, 6.81, and 53638) with different serotypes and different geographic origin (Table 1). To ascertain if and to what extent the polyamine content of these strains had been modified as compared to *E. coli*, their commensal ancestor, we assayed, by means of HPLC, the intracellular level of the major polyamines (spermidine, putrescine, cadaverine, spermine, N-acetylspermidine and N-acetylspermine) when cells were grown in a polyamine-free medium. The polyamine profiles obtained have been compared also with those of *S. flexneri* M90T (Table 1), which has lost cadaverine and N-acetylspermidine during the evolutionary transition from non-pathogenic *E. coli* [22], and of *E. coli* K-12 MG1655.

**Figure 1:**
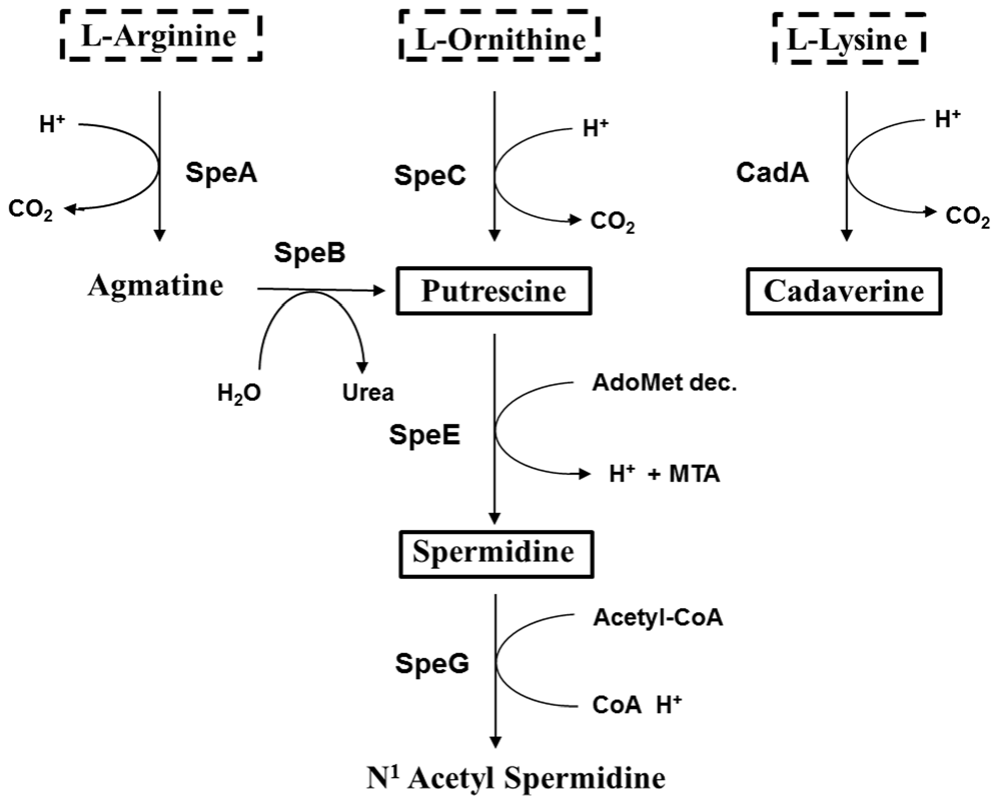
Major steps of the polyamine metabolism in *E. coli*. The diagram depicts the pathway of polyamine biosynthesis in *E. coli*. Data are drawn from Ecocyc database [39]. Dotted boxes: precursor aminoacids. AdoMet dec: S-Adenosyl-L-Methioninamine. MTA: S-Methyl-5′-Thioadenosine.

As reported in Table 2, N-acetylspermidine, the inert form of spermidine obtained by spermidine acetylation (Fig. 1), is still present in all EIEC strains. Strain 13.80 shows a level of N-acetylspermidine comparable to *E. coli* K-12 MG1655. In strains 53638, 6.81, and 4608 N-acetylspermidine is 3.0- to 3.3-fold higher than in *E. coli* K-12 MG1655, while strain HN280 exhibits a 1.8-fold reduction. Statistical analysis shows that these differences are significant (Table 2). As far as intracellular spermidine is concerned, all EIEC strains attain a higher level as compared to *E. coli*. In particular, the increase in strains 13.80, HN280 and 53638 (1.2- to 2.8-fold) is significant (p<0.05), and the higher levels found in strains 4608 and 6.81 are slightly below the significance threshold (p = 0.051 and 0.058 respectively). When referencing the spermidine levels of EIEC to *Shigella*, a significant reduction shows up in all EIEC (1.9- to 2.3-fold; p<0.05) except in strain 53638, where the level remains comparable. It is worth stressing that *Shigella spp.* has faced a complete loss of the *speG* gene product [22]. Interestingly, also the level of putrescine is significantly increased (2.4- to 4.5-fold, p<0.01) in EIEC as compared to *E. coli* while it is comparable to that observed in *Shigella*. In agreement with previous findings [23], [33], we observe that cadaverine is absent from EIEC and *Shigella* spp. Finally, endogenous spermine and acetylspermine are not found in EIEC and *Shigella* (data not shown), which is not surprising since it has been reported also in *E. coli* [24], commonly considered as the commensal ancestor of both *Shigella* and EIEC [16].

**Table 2:** Analysis of polyamine content in different EIEC *and S. flexneri* strains.

	Strain	NSPD	PUT	CAD	SPD
***E. coli*** ** K-12**					
	**MG1655**	5.06±0.41	4.59±0.38	11.2±0.69	3.14±0.35
**EIEC**					
	**13.80**	5.43±0.43	12.23±1.03**	nd	3.89±0.17*
	**HN280**	2.80±0.27*	11.04±1.00**	nd	4.68±0.22*
	**4608**	16.64±1.22**	15.20±1.12**	nd	3.90±0.12
	**6.81**	15.88±1.34**	14.98±1. 16**	nd	3.85±0. 13
	**53638**	15.23±1.43**	20.88±1.99**	nd	8.90±0.74**
***S. flexneri***					
	**M90T**	nd	12.33±2.10**	nd	8.96±0. 68**

Values reported are in nmol per 10^8^ cells and represent the average ± standard deviations. *E. coli* K-12 MG1655 has been used as reference. N-SPD: Acetyl spermidine; PUT: Putrescine; CAD: Cadaverine; SPD: spermidine. Student's t tests were performed comparing PUT, SPD, NSPD and CAD concentration in EIEC and *Shigella* strains with the polyamine concentration detected in *E. coli* K12 MG1655.

*denotes 0.05>p≥0.01;

**denotes p<0.01.

Altogether, our observations indicate that in EIEC, the levels of intracellular putrescine is increased and the levels of spermidine tend to be higher as compared to *E. coli* K-12 MG1655. Moreover, N-acetylspermidine is still present in all strains, in contrast with *Shigella*. This suggests that the polyamine content of EIEC strains has settled at a level intermediate between *Shigella* and *E. coli* K-12 and that the lack of N-acetylspermidine, with the consequent spermidine accumulation, is a trait limited to *Shigella* spp.

### Genetic and functional analysis of the *speG* gene in EIEC

**Figure 2:**
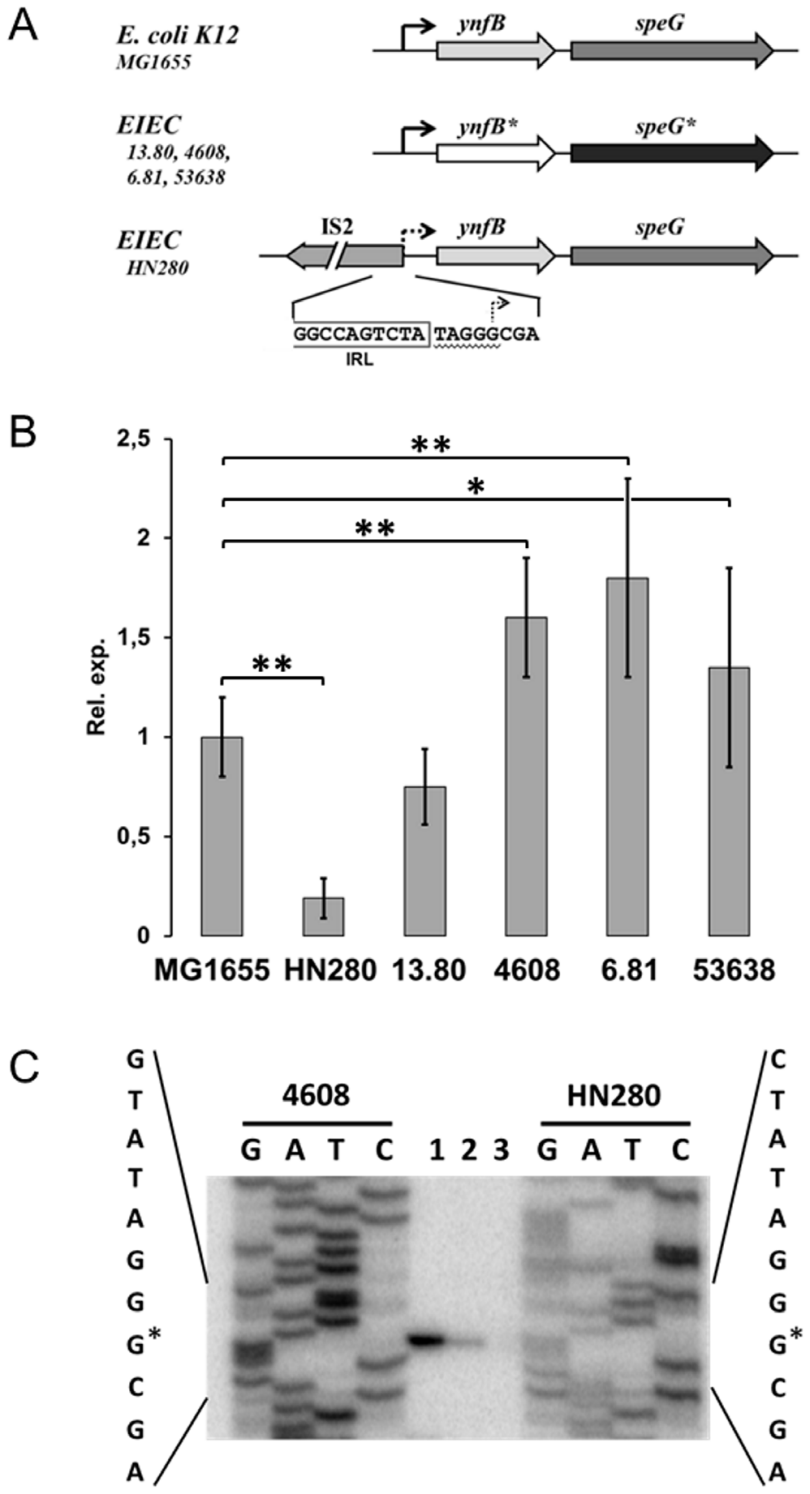
Molecular and functional analysis of the *ynfB-speG* operon in EIEC. (A) Genetic organization of the *ynfB-speG* operon in EIEC strains. The operon at the top is based on the *E. coli* K-12 MG1655 sequence as reported in (National Center of Biotechnology Information, Bethesda, Md.). The right-pointing arrow marks the transcription start site (+1). The asterisk (*) indicates genes carrying non-synonymous mutations. The nucleotide sequence at the bottom refers to the IS*2* insertion into the *ynfB-speG* promoter of EIEC HN280 (IRL: left-end inverted repeat of IS*2*. Underlined: direct repeat at the IS*2* insertion site). (B) Relative *speG* transcription in EIEC strains as monitored by *in vivo* Real-Time PCR using MG1655 as a control. Strains were grown at 37°C in M9 complete medium. At least three wells were run for each sample and the error bars display the calculated maximum (RQMax) and minimum (RQMin) expression levels that represent standard error of the mean expression level (RQ value). * denotes 0.05>p≥0.01; ** denotes p<0.01. (C) Primer extension analysis of the transcripts generated under the control of the *ynfB-speG* promoter of EIEC strains HN280, 13.80, and 4608. The autoradiograph is the result of a typical experiment performed with the *peg* primer on RNA extracted from strain 4608 (lane 1), 13.80 (lane 2), and HN280 (lane 3). Lanes G, A, T, and C show the sequencing ladder generated with the same primer. The asterisk marks the transcription start site (+1).

To understand whether the different levels of N-acetylspermidine observed in our EIEC strains (Table 2) depend on mutations or rearrangements in the *speG* gene promoter, we cloned the corresponding operon (*ynfB-speG*) into pGEM-T Easy, obtaining plasmids pG280, pG13.80, pG4608 pG6.81 and pG53638. PCR amplification, performed using a primer pair (*pgf*/*ygt*) flanking the entire operon, shows that only HN280 gives rise to a product larger than the amplicon of the control strain (MG1655), suggesting the presence of an IS element. Sequence analysis confirms that HN280, which exhibits a reduced level of N-acetylspermidine (Table 2), harbours an IS*2* element inserted within the promoter at position -4 from the transcription start site (+1) (Fig. 2A). Moreover, sequence data (Table S2) reveal that EIEC strains other than HN280 carry a TA transversion within the *ynfB-speG* promoter (position -28), as well as an AC transversion upstream (position -111) the transcription start site. Since these transversions are found in all strains having an N-acetylspermidine level higher than, or comparable to, the *E. coli* K-12 MG1655 control strain, it is possible to speculate that these point mutations are not responsible for the different expression of *speG*.

Also in the case of the *ynfB* gene, located upstream *speG* and encoding a protein of unknown function, a non-synonymous mutation, responsible for a I14F substitution, is present in all strains. Two additional mutations, determining a T25A and a M49I substitution in the YnfB protein, are present only in EIEC 13.80 (Table S2). Except for the presence of the IS*2* element, the HN280 *ynfB-speG* sequence perfectly matches that of *E. coli* K-12 MG1655. Altogether, these data suggest that in EIEC the *ynfB-speG* operon did not undergo the severe rearrangements/deletions seen in *Shigella* [22]. However, the presence of an IS*2* element in the HN280 *ynfB-speG* promoter could represent a symptom of nascent pathoadaptive evolution of the operon.

In order to correlate the amount of N-acetylspermidine observed in our EIEC strains (Table 2) with the expression of the *speG* gene, we monitored the transcription of *speG* in its native background by means of Real Time PCR. As compared to the control, *speG* transcription is strongly activated in EIEC strains 4608, 6.81, and 53638. While the *speG* transcript of strain 13.80 attains a level comparable to that of the control, *speG* transcription in HN280 is severely (5-fold) reduced (Fig. 2B). A possible explanation for this reduction is that the presence of the IS*2* element very close to the transcription start site hampers the transcription of the *ynfB-speG* operon. In order to verify this hypothesis, we analysed, by means of primer extension experiments, *ynfB-speG* transcription in strains HN280, 13.80 and 4608, chosen as representative of the diverse levels of *speG* expression. The results (Fig. 2C) fully agree with the Real Time PCR analysis (Fig. 2B) and confirm that the transcription start site of the EIEC 4608 and 13.80 *ynfB-speG* operon corresponds to the one previously predicted in *E. coli* K-12 [39]. No signal is detected for strain HN280 (Fig. 2C, lane 3), indicating that the IS*2* insertion destroys the promoter and suggesting that the residual expression observed in Real Time PCR assays (Fig. 2B) may be due to read-through from unspecific upstream sites.

**Figure 3:**
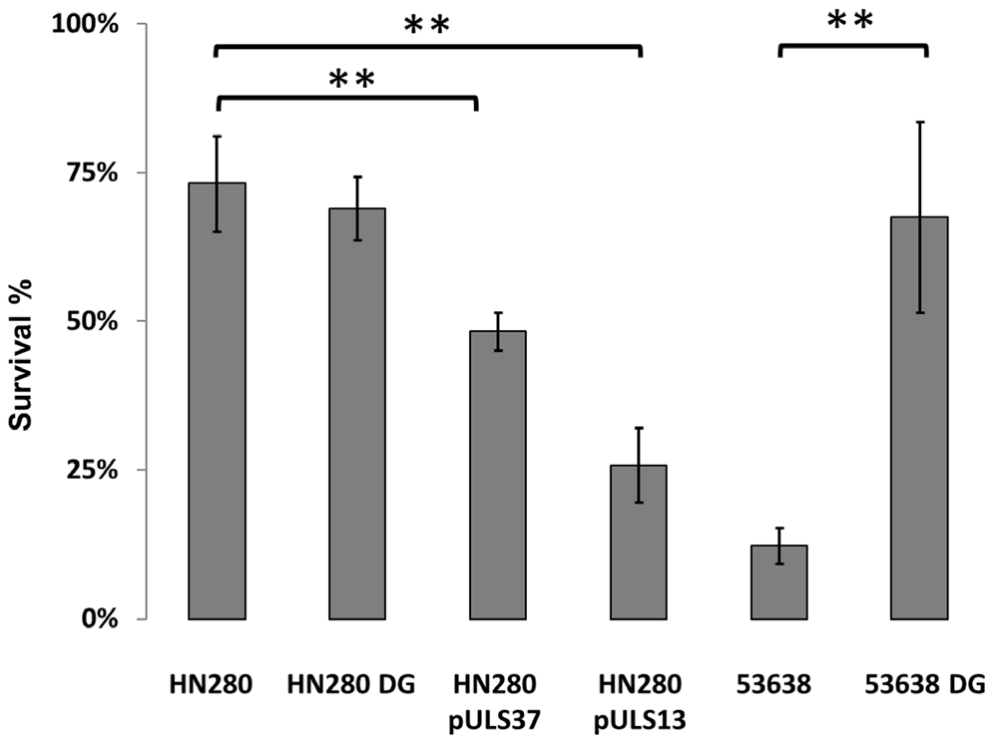
Effect of *speG* on the sensitivity to oxidative stress. The sensitivity to H_2_O_2_ was assayed on EIEC strain HN280 (wild type), on its *speG*-deleted derivative HN280DG, on HN280 complemented with a plasmid carrying *speG* under the control of its own promoter (pULS37) or of the P*tac* promoter (pULS13), on strain 53638 (wild type), and on its *speG* defective derivative 53638 DG. Survival in the presence of H_2_O_2_ was measured after exposure of strains, grown in M9 complete medium, to H_2_O_2_ for 1 h at 37°C. The survival percentage is relative to growth without exposure to H_2_O_2_. Error bars indicate the standard deviations relative to at least three independent experiments. ** denotes p<0.05.

To understand whether the IS*2*-mediated silencing of *speG* confers on EIEC HN280 a selective advantage in response to oxidative stress, we compared the survival of HN280 (wt), the derivatives carrying a plasmid with *speG* under the control of its native promoter (pULS37) or of the P*tac* promoter (pULS13) and, as a control, the HN280 derivative carrying the deletion of the entire *speG* gene (HN280DG). While HN280 and HN280DG respond in a similar manner to the presence of H_2_O_2_, restoration of SpeG activity in HN280 reduces resistance to oxidative stress, and the reduction is directly correlated to *speG* expression since it is further enhanced when *speG* is under the control of a strong promoter (pULS13) (Fig. 3). Additional evidence about the capacity of SpeG to negatively affect resistance to H_2_O_2_ was obtained by studying an EIEC strain (53638) which harbours a functional *speG* gene. The data in figure 3 evidence that under oxidative stress a 53638 *speG* defective derivative (53638 DG) has a higher survival rate as compared to the parental strain.

Finally, to verify whether the lack of the *speG* gene confers a selective advantage during infection in EIEC, we performed an *in vitro* competitive assay analyzing the survival within macrophages of strain HN280 transformed with a plasmid harbouring/lacking a functional copy of *speG*. To this end, HN280 pULS13 and HN280 pACYC184 were grown to OD600 0.6–0.7, mixed and used to infect a murine macrophage cell line (J774). Bacterial survival was monitored during two hours after infection by lysing the macrophages and plating appropriate dilutions on LB plates. The resulting competitive index (ratio of survival rates HN280 pULS13/HN280 pACYC184) corresponds to 0.52 at 1 h and 0.41 at 2 h, indicating that the absence of *speG* enhances survival in macrophages, in agreement with our previous findings in *Shigella* [22]. All together these observations confirm that also in EIEC the absence of SpeG activity confers an increased capability on the bacterium to defy antagonistic host environments.

### Interplay between spermidine and cadaverine production

**Figure 4:**
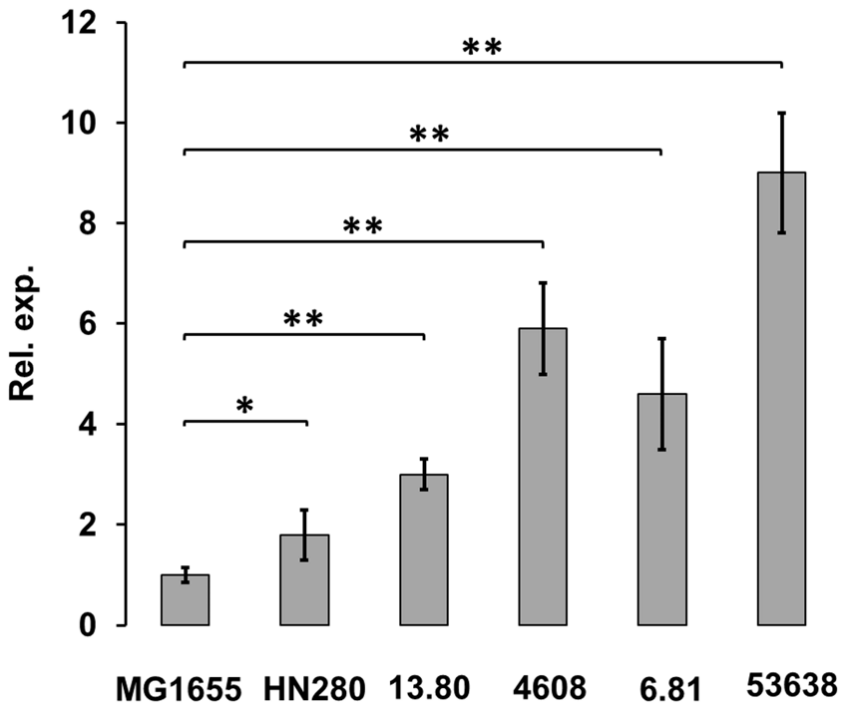
Expression of *speC* in EIEC strains. The *in vivo speC* transcription was monitored by Real-Time PCR and referred to the level observed in the MG1655 control. Strains were grown at 37°C in M9 complete medium. At least three wells were run for each sample and the error bars display the calculated maximum (RQMax) and minimum (RQMin) levels that represent standard error of the mean expression level (RQ value); * denotes 0.05>p≥0.01; ** denotes p<0.01.

Putrescine is found to be approximately 2 to 4-fold higher in EIEC than in *E. coli* K-12 MG1655 (Table 2). Putrescine production (Fig. 1) results from direct ornithine decarboxylation, mediated by the SpeC decarboxylase, and from arginine decarboxylation followed by agmantine ureohydrolyzation determined by the SpeA and SpeB proteins, respectively [24]. To verify whether the increased level of putrescine in EIEC might depend on increased transcription of *speA*, *speB* or *speC*, we monitored mRNA levels in their native backgrounds by Real Time PCR. While no difference is observed in *speA* and *speB* transcription between EIEC and MG1655 strains (data not shown), *speC* transcription is always higher, the increase spanning from 2- to 9-fold. (Fig. 4).

To further clarify this issue, we considered that, despite their genetic and biochemical heterogeneity, all EIEC strains lack lysine decarboxylase and are therefore unable to produce cadaverine [33]. In *E. coli*, the synthesis of cadaverine depends mainly on the gene products of the *cad* locus, which includes three genes, *cadA*, *cadB* and *cadC* [28]. The *cadBA* operon, encoding lysine decarboxylase (CadA) and a lysine cadaverine antiporter (CadB), is submitted to the control of CadC, an integral inner membrane protein which acts both, as signal sensor and as a positive transcriptional regulator. Besides positively regulating the *cadBA* operon, CadC has been shown to negatively interfere with the synthesis of the argine-dependent acid-resistance system [36]. A previous analysis [33] of the major event triggering the loss of cadaverine synthesis in the five EIEC strains used in this study has highlighted that in strains HN280, 4608 and 53638 the *cadBA* operon is integer and that its silencing depends exclusively on the absence of a functional CadC. On the contrary, more severe rearrangements have occurred in EIEC 13.80, where the entire *cad* locus has been lost, and in EIEC 6.81, where the *cadC* gene is inactivated by an IS*1* insertion and the *cadBA* operon is affected by a frameshift mutation in the *cadB* gene [33] (Table 1).

Taking advantage of these *cad*-silencing mutations, we verified whether the expression of the *speC* gene is affected by cadaverine or by the presence/absence of CadC. To this end, we introduced a functional *cadC g*ene (plasmid pCC55) into the five EIEC strains studied and compared the polyamine levels of strains containing or lacking a functional *cadBA* operon. The results (Table 3) indicate that in strains HN280, 4608, 53638 (where the ectopic *cadC* gene is able to restore cadaverine production because of the presence of an integer *cadBA* operon) the amount of intracellular putrescine is severely reduced (about 3-fold). A similar reduction in the putrescine level is observed also in the *E. coli* K-12 control strain ULS655 (MG1655Δ*cadC*) when cadaverine synthesis is restored by the introduction of pCC55. On the contrary, in strains 13.80 and 6.81, which do not contain a functional *cadBA* operon, the level of putrescine is only slightly affected by the presence of CadC. These findings rule out that the CadC regulator is involved in the control of the *speC* gene and strongly suggest that the absence of cadaverine in EIEC has led to increased synthesis of putrescine.

**Table 3:** Analysis of the polyamines levels in EIEC strains complemented or not with a functional copy *cadC* gene.

Strain	*cadC*	NSPD	PUT	CAD	SPD
**ULS655**	-	5.47±0.30	6.75±0.33	0	2.92±0.23	
**ULS655**	+	4.38±0.56	3.40±0.17**	21.15±1.02**	3.12±0.30	
**13.80**	−	5.21±0.26	11.46±0.55	0	3.03±0.19	
**13.80**	+	7.08±0.33*	13.14±0.66*	0	2.96±0.15	
**HN280**	−	3.18±0.12	13.64±0.68	0	4.27±0.15	
**HN280**	+	4.88±0.24*	4.60±0.21**	15.15±0.75**	3.99±0.15	
**4608**	−	16.82±0.84	14.80±0.74	0.00	3.22±0.13	
**4608**	+	15.24±0.76	3.85±0.18**	19.27±0.96**	2.04±0.09**	
**6.81**	−	16.73±0.84	15.71±0.78	0	3.96±0.14	
**6.81**	+	18.90±0.93*	18.87±0.94*	0	4.03±0.20	
**53638**	−	14.19±0.71	21.92±1.01	0	9.08±0.45	
**53638**	+	20.28±0.99*	7.82±0.39**	21.91±1.05**	5.29±0.33**	

Values reported are in nmol per 10^8^ cells and represent the average ± standard deviations. *E. coli* ULS655 (MG1655 *cad*C) has been used as reference. N-SPD: Acetyl spermidine; PUT: Putrescine; CAD: Cadaverine; SPD: spermidine. *cadC*+: strain carrying the pCC55 plasmid; *cadC* -: strain carrying the pACYC184 vector. Student's t tests were performed comparing PUT, SPD, NSPD and CAD concentrations in CadC complemented EIEC strains with the polyamine concentration detected in the corresponding wt (*cadC* ^−^) strains.

*denotes 0.05>p≥0.01;

**denotes p<0.01.

### Cadaverine negatively interferes with SpeC expression

**Figure 5:**
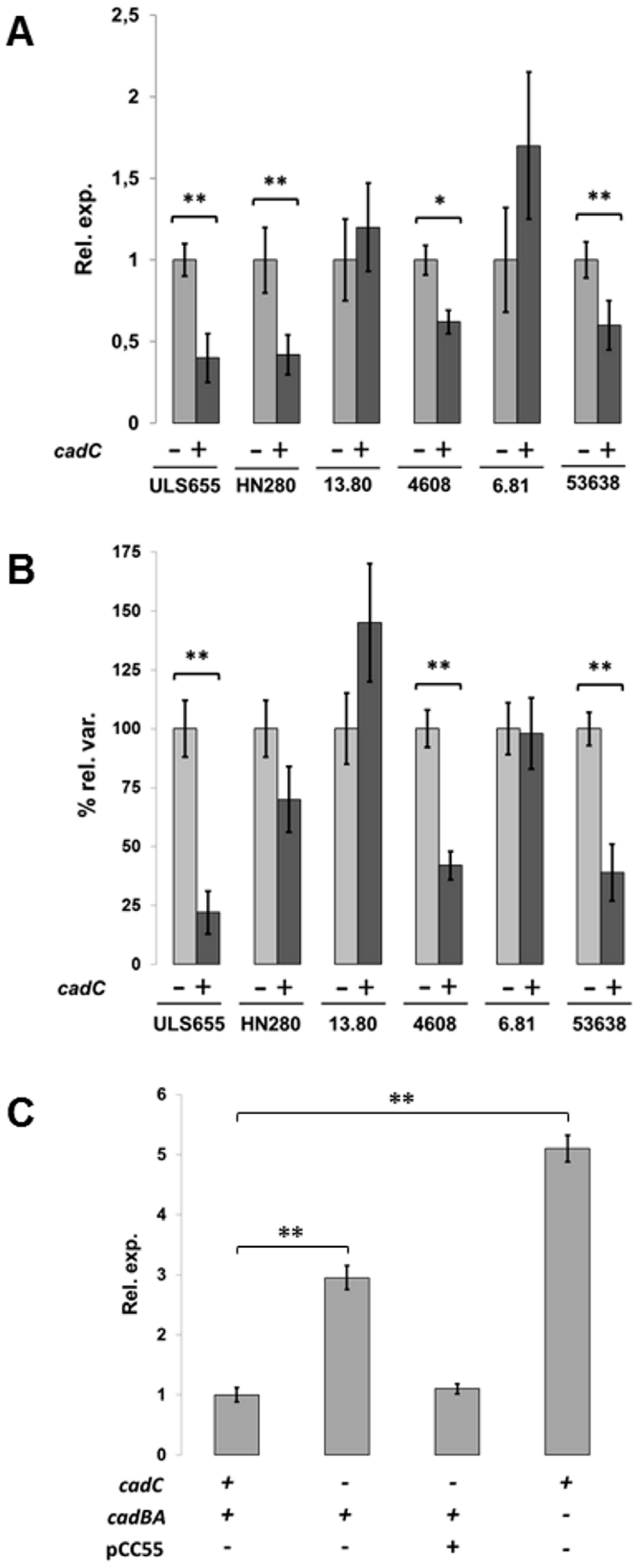
Cadaverine interferes with *speC* expression. Experiments were performed using strains lacking only the *cadC* gene (EIEC HN280, 4608 and 53638 and *E. coli* K-12 ULS655) or the entire *cad* operon (EIEC 13.80 and 6.81). Strains were transformed with pCC55 (a pACYC184 derivative containing a functional *cadC* gene) or pACYC184. (A) Transcription of *speC* in the presence or absence of cadaverine as monitored by Real-Time PCR. At least three wells were run for each sample and the error bars display the calculated maximum (RQMax) and minimum (RQMin) levels that represent standard error of the mean expression level (RQ value). * denotes 0.05>p≥0.01; ** denotes p<0.01 (B) Ornithine decarboxylase (ODC) activity in the presence or absence of cadaverine. ODC activity was measured in total cell extracts by assaying putrescine production. The synthesized putrescine was made fluorescent through chemical modification and separated by TLC. The fluorimetric data were normalized against the total protein content of each sample. Results are shown as variation (percentage) in strains carrying pCC55 (*cadC*) vs the corresponding wt strain carrying the backbone construct. * denotes 0.05>p≥0.01; ** denotes p<0.01. (C) Transcription of *speC* in *E. coli* K-12 (MG1655); ** denotes p<0.01. Transcription in strains producing or lacking cadaverine was monitored by Real-Time PCR as in panel A.

To find out how strictly the expression of SpeC depends on cadaverine, we analysed *speC* transcription in EIEC strains and in the ULS655 control strain complemented with a functional *cadC* gene (pCC55). Comparative analysis performed by Real Time PCR (Fig. 5A) indicates that *speC* transcription is strongly reduced in ULS655 and in EIEC HN280, 4608 and 53638 when cadaverine synthesis is restored by the introduction of a functional *cadC* gene [33]. On the contrary, the presence of CadC does not affect significantly *speC* expression in EIEC strains 13.80 and 6.81, which lack an integer *cadBA* operon. To ascertain whether the observed transcriptional regulation is primarily responsible for SpeC expression and putrescine production, we estimated relative ornithine decarboxylase activity in total cell extracts from EIEC strains HN280, 4608, 53638, 13.80, and 6.81. As shown in Fig. 5B ornithine decarboxylase activity is decreased only in strains which produce cadaverine (HN280, 4608 and 53638) after *cadC*-complementation. This nicely matches with the reduced transcriptional regulation of the *speC* gene in the same strains, shown in Fig. 5A. Furthermore, these data are consistent with the polyamine abundance reported in Table 3 and strongly stress the key role played by cadaverine in modulating the level of putrescine.

In order to check whether the inhibitory effect of cadaverine on *speC* expression also applies to polyamine metabolism in *E. coli*, and to rule out incidental contributions from undefined factors possibly present in the EIEC background, we studied the role of cadaverine in an *E. coli* K-12 background. Exploiting the one-step gene inactivation method [37], we first constructed an *E. coli* MG1655 derivative defective in the *cadBA* operon (ULS86, Table 1). Then, we introduced the pCC55 plasmid into ULS655 (*cadC*) or in ULS86 (*cadBA*). The expression of the *speC* gene was monitored by Real Time PCR. The results (Fig. 5C) show that in a *cadC* defective background (ULS655) the level of *speC* transcription is 3-fold higher as compared to wt (MG1655) and to *cadC* complemented ULS655 strain, and that in a *cadBA* defective background (ULS86 pCC55) the increase is even higher (5-fold). These data strongly support the hypothesis that the higher level of putrescine observed in EIEC strains is a consequence of the evolutionary loss of the *cad* operon.

## Discussion

Polyamines are small polycationic molecules found in eukaryotic and prokaryotic cells, associated with a wide variety of biological functions. Putrescine, cadaverine, spermidine and spermine are the major polyamines in bacteria [24]. Their intracellular content is regulated by the concerted action of biosynthesis and uptake processes, as well as degradation and efflux mechanisms. Synthesis usually depends on the decarboxylation of precursor aminoacids (or other intermediates) which are then converted into functional polyamines. Trafficking relies mainly on uptake and exchange processes mediated by specific ABC transporters and antiporters [25]. In recent years, it has become increasingly evident that, in addition to core physiological functions, polyamines play an active role in bacterial virulence [22], [23], [47], [48], [49]. Several strategies have been developed by bacterial pathogens to exploit polyamines or manipulate polyamine-related processes to optimize bacterial fitness within the host. Some bacterial pathogens are able to utilize polyamines to favour their survival within the host or to alter the immune response of the host. In other cases, polyamines are crucial to promote the expression of virulence factors, as T3SS, or to activate biofilm formation [26], [27].

The essential role played by polyamines in bacterial virulence is nicely exemplified by *Shigella*, an intracellular pathogen which belongs to the *E. coli* species and causes a severe enteric syndrome in humans [22]. *Shigella* evolved from its innocuous ancestor, *E. coli*, through several steps, which include a gain of functions facilitating the intracellular survival and loss of functions hampering the full expression of an invasive phenotype [7], [11], [16]. While the acquisition of the large virulence plasmid (pINV) by the *Shigella*/EIEC pathotype has induced, in a single step, the capacity to enter and multiply inside the highly specialized intracellular environment of the human intestinal mucosa [5], [8], [17], the loss of antivirulence functions (pathoadaptive mutations) has acted progressively to increase the pathogenic potential of these strains [6], [7]. Among pathoadaptive mutations, a paradigmatic case is represented by the inactivation of genes involved in the biosynthesis of polyamines. In particular, as compared to the commensal *E. coli*, *Shigella* has completely lost cadaverine [23], [30], [31] and N-acetylspermidine, (the inert form of spermidine), and displays a marked accumulation of spermidine [22].

The silencing of genes involved in the biosynthesis of cadaverine is a key factor in the optimization of the pathogenicity process of *Shigella*, since secreted cadaverine blocks the release of the bacterium into the cytoplasm of infected cells by stabilizing the endosomal membrane and negatively affects *Shigella*-induced proinflammatory events by inhibiting PMN migration to the infection site [6]. The increased spermidine content of *Shigella* depends on the lack of a functional *speG* gene, i.e. on the absence of spermidine acetyltransferase (SAT), the enzyme which converts spermidine into N-acetylspermidine [50]. A distinct advantage results: higher spermidine levels have been shown [22] to increase survival within macrophages during the initial step of the infection process. Enteroinvasive *E. coli* (EIEC) share with *Shigella* the same infective process and, as for genetic and phenotypic features, are considered evolutionary intermediates between the harmless *E. coli* and the harmful *Shigella* [10], [12]. Similarly to *Shigella*, also EIEC have acquired the pINV virulence plasmid and have undergone pathoadaptation starting from their ancestor [5]. While the lack of cadaverine has been extensively analysed in EIEC [33], [36], so far no data concerning the presence of the other polyamines were available.

The results we obtained in this study indicate that the polyamine content of EIEC is intermediate between *E. coli* and *Shigella*. Indeed, intracellular putrescine is significantly increased in EIEC while spermidine tends to be higher as compared to *E. coli* K-12. However, N-acetylspermidine is still present in most strains we have analysed, indicating that the loss of *speG* as pathoadaptive mutation is an emerging, albeit not fully acquired, trait of EIEC. In particular, in four out of five EIEC strains we have analysed, the *speG* gene is expressed at a level comparable or higher than in the *E. coli* K-12 control, whereas only one EIEC strain (HN280) displays a severe reduction of N-acetylspermidine (Table 2). We have shown that, a few bases upstream the transcription start site, the promoter of the HN280 *ynfB-speG* operon harbours an IS*2* element which is likely responsible for silencing the *speG* gene (Fig. 2A). The residual *speG* transcription in HN280 is likely the result of read-through transcription, since primer extension analysis does not reveal any signal in correspondence to the expected transcription start base (Fig. 2C).

As for the increased expression of *speG* in EIEC stains 4608, 6.81 and 53638 (Fig. 2B), our observations indicate that it does not depend on the presence of two transversions within the promoter region (positions -28 and -111), since these base changes (Table S2) are found also in EIEC strain 13.80, where *spe*G transcription is comparable to that observed in the *E. coli* K-12 control (Fig. 2B). Therefore, it is reasonable to assume that the level of *speG* transcription detected in these strains may be due to the increased spermidine biosynthesis elicited by the higher putrescine content. In particular, this is evidenced by EIEC 53638 where putrescine and spermidine attain the highest levels among the strains investigated.

It is not surprising that the lack of *speG* (and the consequent accumulation of spermidine) is not a common feature of EIEC strains, since EIEC do not constitute a homogeneous group of pathogenic *E. coli*. Indeed, phylogenetic studies of housekeeping genes reveal that EIEC do not belong to a single *E. coli* cluster and have several distinct evolutionary origins, i.e they emerged in several independent events from multiple ancestral *E. coli* [4], [10], . EIEC possess many *Shigella*-like features, however they do not have the full set of characters that define *Shigella* strains and are not included in any of the three *Shigella* clusters. In contrast to *Shigella*, EIEC have a high metabolic activity since they still retain the ability to catabolize substrates widely utilized by *E. coli* [14]. It is still an open question whether EIEC strains should be regarded as ancestral forms which are developing towards *Shigella* or as forms that have acquired a *Shigella*-type adaptation to the human host but are better equipped to face the challenges of the outer environment.

Interestingly, the evolutionary analysis of the polyamine content of EIEC we present in this study underscores the interplay between cadaverine and the expression of the *speC* gene, encoding ornithine decarboxylase. As one of the major activities in the polyamine pathway this enzyme is responsible for an essential step, the conversion of the L-ornithine into putrescine (Fig. 1). Our data show that the absence of cadaverine in EIEC, depending on the silencing of the *cad* operon brought about by convergent evolution [29], is responsible for increased *speC* transcription and, consequently, increased putrescine production. Though the molecular mechanisms adopted by cadaverine to negatively interfere with *speC* expression deserve further investigation to be fully clarified, the crucial role of cadaverine shows up. Indeed, restoring cadaverine synthesis (by introducing a functional *cadC* gene) in EIEC strains harboring a functional *cadBA* operon reduces *speC* expression and intracellular putrescine (Fig. 5C).

**Figure 6:**
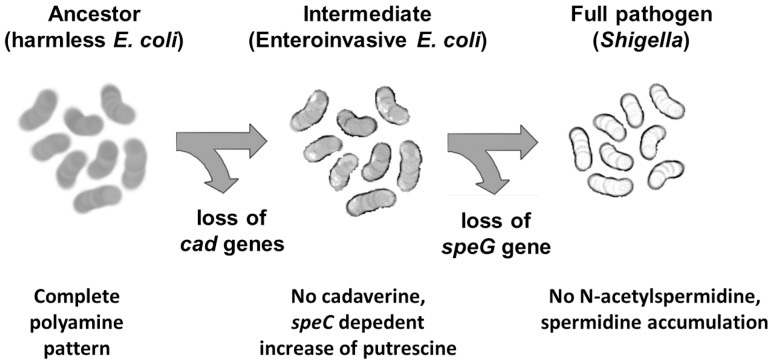
A model of the pathoadaptive evolution of the polyamine profile in EIEC and *Shigella* spp. In this view the loss of *cad* genes marks the transition from the polyamine profile of the commensal *E. coli* ancestor to that of enteroinvasive *E. coli* (EIEC), characterized by lack of cadaverine and increased putrescine content. The loss of the *speG* gene, occurring in a successive evolutionary step and inducing spermidine accumulation (and, consequently, higher resistance to oxidative stress), gives rise to the *Shigella*-type polyamine profile.

On the basis of our observations, it is reasonable to speculate (Fig. 6) that during the transition from a commensal ancestor (*E. coli*) towards an enteroinvasive pathogenic phenotype (EIEC) the modification of the polyamine profile might have been triggered by the loss of cadaverine. It is known that cadaverine negatively interferes with the invasive process [23] and it is likely that it has been lost during the initial steps of the pathoadaptation process. In turn, the lack of cadaverine may have induced an increased putrescine level, as we have observed in our EIEC collection and reproduced in an *E. coli* K-12 background. Since putrescine is a relevant intermediate in the synthesis of spermidine and, consequently, of N-acetyspermidine, an increase in putrescine may well have caused higher levels of both, spermidine and N-acetylspermidine. The inactivation of the *speG* gene would represent the last step, favouring the accumulation of spermidine (and the elimination of N-acetylspermidine) and enhancing the survival in oxidative environments. In this view, the loss of *speG*-function has already occurred in *Shigella* spp. and, banking on our data, could be regarded as an ongoing process in EIEC. In both microorganisms, the lack of *speG* increases the resistance to oxidative stress and confers higher survival within macrophages. Altogether, our observations agree well with the hypothesis that EIEC are an intermediate step during the transition of *E. coli* towards a full-blown *Shigella* phenotype, and further clarify mechanisms and strategies adopted by these bacterial pathogens during the infectious processes.

## Supporting Information

Table S1 **Oligos used in this study.**
(DOC)

Table S2 **List of the point mutations (transitions and transversion) found in the EIEC** ***ynfB-speG*** **locus and its promoter.**
(DOC)
