# Supplementary material for: Molecular and Functional Profiling of the Polyamine Content in Enteroinvasive E. coli : Looking into the Gap between Commensal E. coli and Harmful Shigella
Source: PLoS One. 2014 Sep 5;9(9):e106589. doi: 10.1371/journal.pone.0106589 (PMC4156367; doi:10.1371/journal.pone.0106589)
Supplement: Table S1 — Oligos used in this study. (DOC) [file pone.0106589.s001.doc]

**Table S1: Oligos used in this study**

| **Oligos** | **Sequence 5’- 3’** |
| --- | --- |
| *caf* | ATGAGTTCTGCCAAGAAGATCGGGCTATTTGCCTGTGTGTAGGCTGGAGCTGCTTC |
| *car* | TTATTTTTTGCTTTCTTCTTTCAATACCTTAACGGTATTCCGGGGATCCGTCGACC |
| *dgf* | GAAGCCTTTGTTTGAACTCTCTGATCTGTATGATAAGCGTGTAGGCTGGAGCTGCTTCG |
| *dgr* | ATAGAGATTGAGAACGGTAAAGCCATAGTCCATTGCATTCCGGGGATCCGTCGACC |
| *pgf* | NGAATTCCCACAACCAGGGCAGAAAGCA |
| *ygt* | GAAGGAGTAAAACCCGCCGTC |
| *rgf* | AACGCCAGTGTGATGCGTTA |
| *rgr* | CAGAGAGTTCAACAAAGGCTTCGT |
| *scf* | TGCTGTTGAATTACCTGCGG |
| *scr* | TGCTCGTTGCCGTTGATTAC |
| *nusAF* | TGAAGCCGCACGTTATGAAG |
| *nusAR* | TCAACGTAATTCGCCCAGGTT |
| *peg* | GCAAGGCAGCAGAAAAGCGAGC |
