# Supplementary material for: Molecular and Functional Profiling of the Polyamine Content in Enteroinvasive E. coli : Looking into the Gap between Commensal E. coli and Harmful Shigella
Source: PLoS One. 2014 Sep 5;9(9):e106589. doi: 10.1371/journal.pone.0106589 (PMC4156367; doi:10.1371/journal.pone.0106589)
Supplement: Table S2 — List of the point mutations (transitions and transversion) found in the EIEC ynfB-speG locus and its promoter. (DOC) [file pone.0106589.s002.doc]

Tabella S2 List of the point mutations (transitions and transversion) found in the EIEC *ynfB-speG* locus and its promoter

|  | **EIEC strain** | **transition** | **transversion** |
| --- | --- | --- | --- |
| **Promoter**  **region** |  |  |  |
|  | 6.81 | AG -86 | TA -28 |
|  | 13.80 |  | TA -28 |
|  | 4608 |  | TA -28 |
|  | 53638 |  | TA -28 |
| ***ynfB*** |  |  |  |
|  | 6.81 | AT139 | CA390 |
|  | 13.80 | AT139; TC163; AG172; CT282 | GC246; CA390 |
|  | 4608 | AT139 | CA390 |
|  | 53638 | AT139; TC163; CT282 | CA390 |
| ***speG*** |  |  |  |
|  | 6.81 | GA649; CT695;TA715; TC811; CT919 | TA896 |
|  | 13.80 | GA649; TA715; TC811; TC847; CT919; CT925 | CG724; TA896 |
|  | 4608 | GA649; CT695; TA715; CT919 | TA896 |
|  | 53638 | GA649; TA715; TC811; TC847; CT919; CT925 | CG724; TA896 |

The underlined transitions and transversions are non-homologous mutations: AT139, AG172 and GC246 gave rise to I14F, T25A and M91I aminoacid substitution in YnfB protein; TA896 gave rise to S141T aminoacid substitution in SpeG protein. The numbering of mutations is relative to the transcriptional starting site of *ynfB speG* operon.
